# Comparison of conventional, amplification and bio-assay detection methods for a chronic wasting disease inoculum pool

**DOI:** 10.1371/journal.pone.0216621

**Published:** 2019-05-09

**Authors:** Erin McNulty, Amy V. Nalls, Samuel Mellentine, Erin Hughes, Laura Pulscher, Edward A. Hoover, Candace K. Mathiason

**Affiliations:** Department of Microbiology, Immunology and Pathology, Colorado State University, Fort Collins, Colorado, United States of America; National Institute of Allergy and Infectious Diseases, UNITED STATES

## Abstract

Longitudinal studies of chronic wasting disease (CWD) in the native host have provided considerable understanding of how this prion disease continues to efficiently spread among cervid species. These studies entail great cost in animal, time and financial support. A variety of methods have emerged including transgenic mouse bioassay, western blot, enzyme-linked immunoassay (ELISA), immunohistochemistry (IHC), serial protein misfolding cyclic amplification (sPMCA) and real time quaking-induced conversion (RT-QuIC), that deepen our understanding of this and other protein misfolding disorders. To further characterize an inoculum source used for ongoing CWD studies and to determine how the readouts from each of these assays compare, we assayed a CWD-positive brain pool homogenate (CBP6) and a mouse dilutional bioassay of this homogenate using the above detection methods. We demonstrate that: (i) amplification assays enhanced detection of amyloid seeding activity in the CWD+ cervid brain pool to levels beyond mouse LD_50_, (ii) conventional detection methods (IHC and western blot) performed well in identifying the presence of PrP^Sc^ in terminal brain tissue yet lack sufficient detection sensitivity to identify all CWD-infected mice, and (iii) the incorporation of amplification assays enhanced detection of CWD-infected mice near the LD_50_. This cross-platform analysis provides a basis to calibrate the relative sensitivities of CWD detection assays.

## Introduction

Chronic wasting disease (CWD) is unique among the prion diseases due to its capacity to infect a wildlife population and for its unparalleled transmission efficiency. Infectious particles shed in several bodily fluids and excretions from infected, yet asymptomatic cervids likely contribute to this efficient transmissibility [1–6]. Additional concern is warranted for cervids and species sympatric with cervid populations as the geographical distribution, host range [7, 8, 9] and strain identification [10–12] of CWD continues to expand, undoubtedly resulting in increased environmental contamination. Although no cases of CWD transmission to humans have been reported, its zoonotic potential remains a concern [10, 13] due to the prevalence of asymptomatic CWD in deer and elk [14, 15] (commonly consumed by humans) and the precedent of BSE transmission to humans causing vCJD [16]. This growing zoonotic concern, in concert with increased import in early detection for all protein misfolding disorders, has led to enhanced efforts to develop novel tools to detect and monitor prion infections earlier in the disease course.

Diagnoses of prion diseases have historically relied upon detection of the biological marker associated with disease (PrP^Sc^) [17] in terminal brain and lymphoid tissues using western blot (WB), immunohistochemistry (IHC) and enzyme linked immunoassay (ELISA) [18]. Although these methods efficiently diagnose prion infections postmortem, they lack sufficient sensitivity for consistent acute phase detection [18]. Enhanced early detection could estimate prion burdens in tissue and biological fluids, and provide new insights into peripheral prion trafficking, disease pathogenesis, and transmission dynamics to help guide the development of non-invasive diagnostics, therapeutic strategies and management practices.

The major challenge to early diagnosis of prion diseases has been the very low prion concentrations and/or inhibitors that are present in accessible biological samples [19–21]. In addition, prion infectivity is comprised of variably protease sensitive particles [22], yet conventional prion detection methods require the use of protease digestion, which ablates sensitive forms of prion infectivity (PrP^Sen^) and reduces sensitivity. Thus, while bioassay in native and transgenic hosts remains the gold standard for assessment of prion infectivity in biological samples, it remains burdensome due to animals, time required and cost.

The emergence of *in vitro* prion amplification detection methods, including serial protein misfolding cyclic amplification (sPMCA) and real-time quaking induced conversion (RT-QuIC), have provided rapid and highly sensitive means for prion detection. Unlike IHC, WB and ELISA, sPMCA and RT-QuIC are seeding assays that rely upon the conversion of the native prion protein (PrP^C^) into PrP^Sc^. sPMCA [23] uses brain homogenate from transgenic mice overexpressing PrP^C^ as substrate, alternating sonication and incubation to amplify PrP^Sc^ [24]. This method has been successful in detecting misfolded prion protein in a variety of tissues and bodily fluids from prion-infected hosts [18, 25]. RT-QuIC is a second *in vitro* amplification assay [26] that has been widely used to detect protein misfolding disorders, including prions [6, 27–32]. RT-QuIC employs recombinant normal prion protein (rPrP^C^) and intermittent shaking and incubation to initiate the templated conversion of PrP to a misfolded amyloid PrP^Sc^-like form [26], detected by the intercalation of thioflavin T(ThT) into growing amyloid fibrils. RT-QuIC has been shown to detect amyloid seeding activity in a variety of biological tissue and fluid samples [6, 33–40]. Importantly, both sPMCA and RT-QuIC are capable of detecting prions during the early asymptomatic phase of disease [40–45].

We have conducted several past and ongoing analyses, including longitudinal studies in the native host, utilizing a single CWD+ brain homogenate pool (CBP6) to garner better understanding of CWD pathogenesis and transmission dynamics. A direct analysis of this pool by the above *in vitro* assays and bioassay has not been conducted. To directly address this gap in characterization and to gain a better understanding of how readouts from these methods compare across a single inoculum, we have assessed: (i) CWD+ cervid brain homogenate pool CBP6, and (ii) brain tissue from a transgenic mouse end-point dilutional bioassay of the same homogenate [36] by *in vitro* assays: WB, ELISA, IHC, sPMCA, RT-QuIC and a combination of sPMCA + RT-QuIC.

## Results

### Prion detection in CWD+ deer brain homogenate (CBP6)

#### Detection of PrP^Sc^ by conventional western blot (WB) and BioRad enzme-linked immunosorbent assay (ELISA)

**Fig 1:**
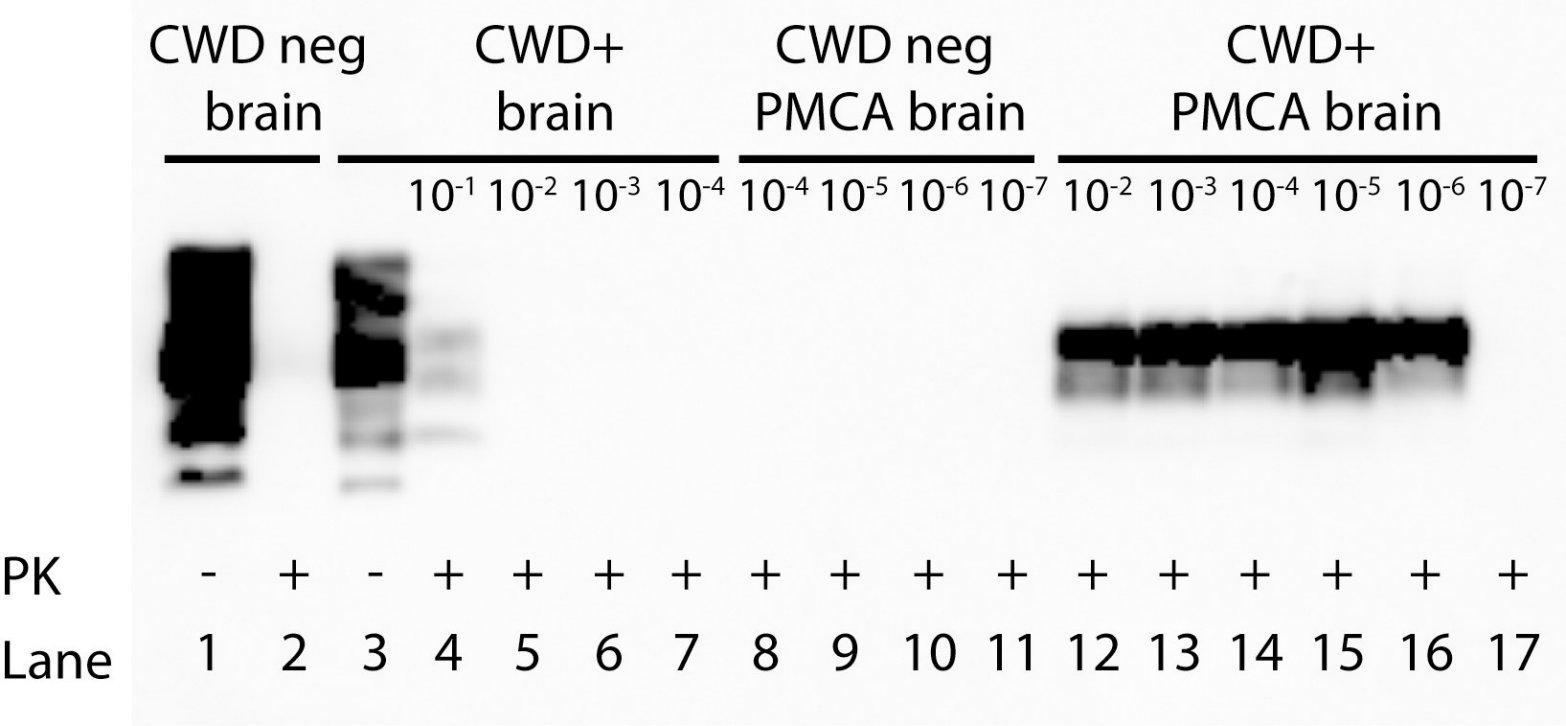
Western blot comparison of prion detection in a dilutional series of unamplified and sPMCA amplified CWD+ cervid brain. Detection of PrP^Sc^ is shown in the 10^−1^ dilution of unamplified CWD+ cervid brain homogenate CBP6 (500 μg; lane 4). No PrP^Sc^ was detected in further dilutions of unamplified CBP6 (lanes 5–7). Detection of PrP^Sc^ is shown in the 10^−2^–10^−6^ dilutions of sPMCA amplified CBP6 (5 rounds PMCA; initiating seed = 20 μg to 2 ng; lanes 12–16), but not in the 10^−7^ dilution (200 pg). Complete PK digestion of PrP^C^ is shown in unamplified (lane 2) and sPMCA amplified naïve deer brain (5 rounds; lanes 8–11).

Previous studies have demonstrated that conventional methods are adequate to detect prions in tissues harvested postmortem [46–49]. As expected, we were able to demonstrate detection of PrP^Sc^ by WB in the 10^−1^ dilution of unamplified CWD+ cervid brain homogenate following proteinase K (PK) digestion. No amyloid was detected in further dilutions of CWD+ cervid brain CBP6 (10^−2^ to 10^−7^), nor in age-matched naïve deer brain (Fig 1, Table 1). BioRad ELISA detected PrP^Sc^ in 10^−1^ and 10^−2^ dilutions (OD readings of 3.999 and 0.259 respectively) of unamplified CWD+ cervid brain pool CBP6. No amyloid was detected in further dilutions (10^−3^–10^−9^), nor in age-matched naïve deer brain (Table 1).

**Table 1:** Comparison of prion detection in a dilutional series of CWD+ cervid brain pool.

	Cervid brain pool homogenate dilution								
Assay	10^−1^	10^−2^	10^−3^	10^−4^	10^−5^	10^−6^	10^−7^	10^−8^	10^−9^
**Western Blot**									
**BioRad ELISA**									
**Mouse Bioassay**	ND					*LD_50_			ND	ND
**sPMCA/WB**								ND	ND
**RT-QuIC**	NT	NA							
**sPMCA/RT-QuIC**	NT								

CWD+ cervid brain pool homogenate (CBP6) was serially diluted and assayed for prion endpoint dilution. Conventional WB detected prions at 10^−1^ (unamplified; 500 μg). BioRad ELISA detected prions at 10^−1^ and 10^−2^ dilutions (unamplified; 3 mg-300 μg. Tg(CerPrP) 5037 mouse bioassay demonstrates prion infectivity within CBP6 dilutions 10^−2^–10^−6^ (300 μg-30 ng). sPMCA/WB detects amyloid seeding activity from 10^−1^–10^−6^ (20 μg-2 ng; 10^−8^–10^−9^ not done (ND)). Amyloid seeding activity is detected by RT-QuIC from 10^−3^–10^−7^ (2 μg-200 pg). RT-QuIC demonstrates no amplification (NA) at dilution 10^−2^ (20 μg). sPMCA/RT-QuIC detects amyloid seeding activity from 10^−2^–10^−8^ (20 μg-20 pg). No assay detected prions at CBP6 dilution 10^−9^. Bioassay not done (ND) at 10^−1^ or 10^−8^–10^−9^. 10^−1^ dilutions were not tested (NT) for RT-QuIC and sPMCA/RT-QuIC due to required dilution into PrP^C^ substrate. Shaded squares indicate prion positivity. *LD_50_ = 10^5.5^.

#### Detection of seeding activity by new generation assays: sPMCA, RT-QuIC or sPMCA/RT-QuIC

We and others [50, 51] have demonstrated that tissues harvested from infected hosts, especially those in the asymptomatic phase of disease, may contain low levels or variably PK resistant forms of the misfolded protein. The presence of very low concentrations of prion deposition diminishes ability to rely solely upon IHC, ELISA or WB for the detection of PrP^Sc^ deposition associated with prion disease. Thus, we assessed and compared endpoint dilution of the same CWD+ cervid brain (CBP6) dilutional series using these assays—sPMCA, RT-QuIC and RT-QuIC readout of sPMCA 5^th^ round product (sPMCA/RT-QuIC).

**Fig 2:**
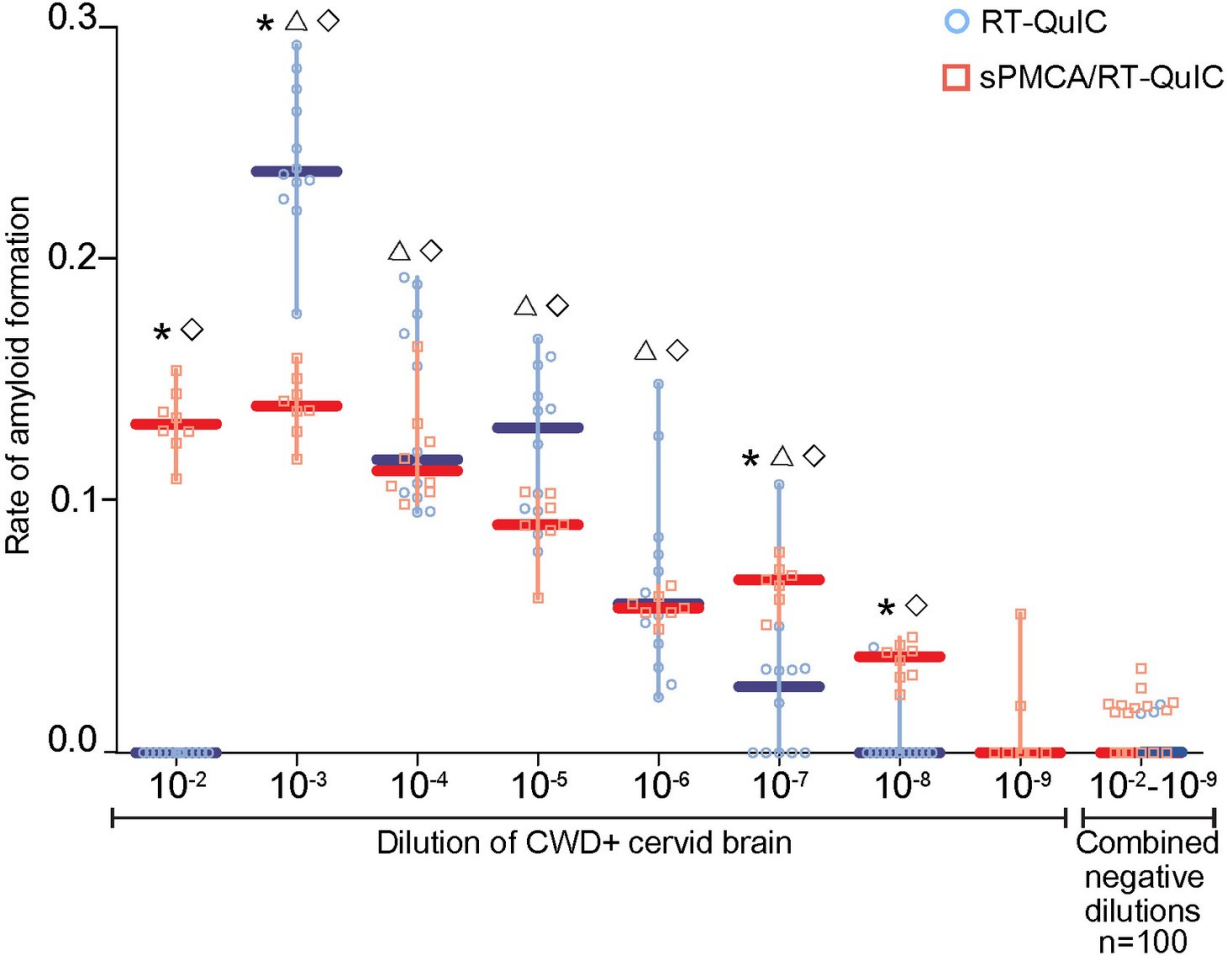
Comparison of amyloid seeding activity in a dilutional series of CWD+ cervid brain by RT-QuIC and sPMCA/RT-QuIC. Amyloid seeding activity was detected by RT-QuIC in the 10^−3^–10^−7^ dilutions of CWD+ cervid brain pool CBP6 (2 μg-200 pg seed material; blue circles and blue median lines). sPMCA/RT-QuIC assay (round 5 sPMCA product diluted 1:100) detected amyloid seeding activity in the 10^−2^–10^−8^ dilutions of CBP6 (20 μg-20 pg seed material; red squares and red median lines). Neither assay detected significant amyloid seeding activity in the 10^−9^ dilution of CBP6 (2 pg seed material) or in naïve cervid brain homogenate dilutions (100 replicates^ϒ^). 7–12 replicates are shown per assay dilution. Statistical significance between assays is indicated with an asterisk (≤ 0.0017). Statistical significance between samples and same dilution negative controls is indicated with Δ RT-QuIC and ◊ sPMCA/RT-QuIC (≤ 0.0001). ^ϒ^negative controls from all dilutions were combined as no dilutional effect was noted.

The conventional readout for prion seeding activity generated by sPMCA is WB (sPMCA/WB). We subjected a dilutional series of naive and CBP6 CWD+ cervid brain homogenate (10^−2^ to 10^−7^) to 5 rounds of sPMCA. When assessed by WB, we revealed the presence of amyloid seeding activity to an endpoint dilution of 10^−6^ (Fig 1, Table 1). Upon assessment of the same CWD+ cervid brain dilutional series by RT-QuIC we determined the endpoint for amyloid seeding activity to 10^−7^ (Fig 2, Table 1). We have recently used RT-QuIC as a readout for sPMCA product [52] (sPMCA/RT-QuIC). Incorporation of RT-QuIC as the readout for sPMCA 5^th^ round product improved detection of amyloid seeding activity in CWD+ cervid brain homogenate to 10^−8^ (Fig 2, Table 1). No amyloid seeding activity was detected at any dilution of the naïve brain homogenate post sPMCA/WB, RT-QuIC or sPMCA/RT-QuIC amplification.

#### sPMCA/RT-QuIC optimum seed input

**Fig 3:**
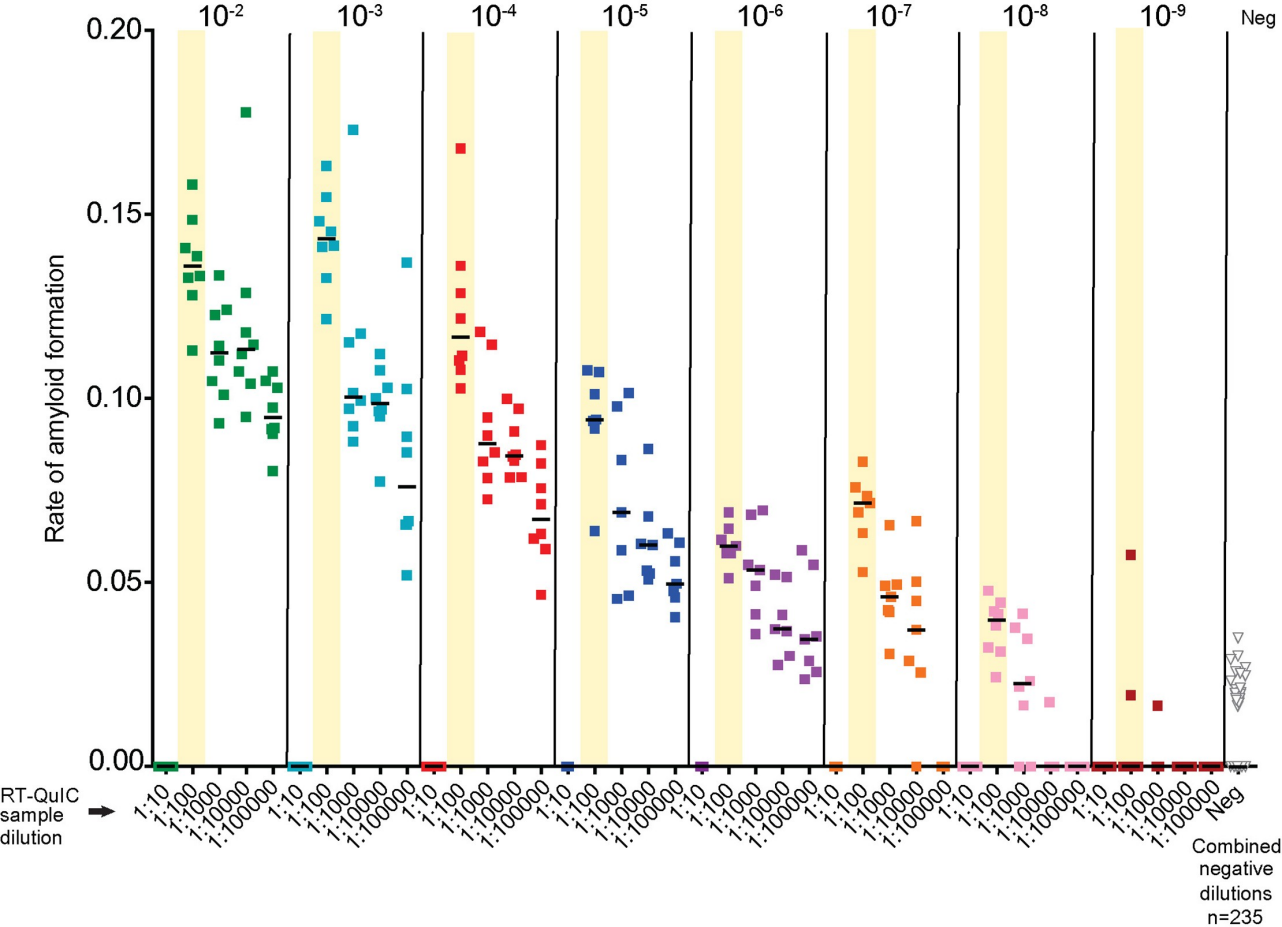
Detection of amyloid seeding activity by sPMCA/RT-QuIC in a CWD+ cervid brain dilutional series. Amyloid seeding activity was detected in 10^−2^–10^−8^ dilutions of CWD+ cervid brain (CBP6) (20ug-20pg) using 1:100–1:100000 dilutions of sPMCA 5^th^ round product. Amyloid seeding activity was inhibited at the 1:10 dilution. Optimal prion detection was demonstrated at a 1:100 dilution (yellow bars) of sPMCA 5th round product. No significant amyloid seeding activity was initiated by 10^−2^–10^−9^ dilutions of naïve cervid brain using 1:100–1:100000 dilutions of sPMCA 5^th^ round product. n = 7–8 RT-QuIC replicates/sample. n = 235 negative control dilution replicates.

RT-QuIC amyloid seeding activity can be inhibited at high seed concentrations and rescued by dilution [5, 53, 54]. To establish the optimal seed input for maximal amyloid seeding detection by sPMCA/RT-QuIC we assessed a dilutional series of CBP6 5^th^ round sPMCA product by RT-QuIC (Fig 3). We demonstrate that RT-QuIC amyloid seeding activity is rescued when 5^th^ round sPMCA CBP6 is diluted beyond a 1:10 dilution (Fig 3). We also reveal maximal readout for the combined assay when CBP6 sPMCA 5^th^ round product (initiated by 1:1000 dilution) is spiked at 1:100 dilution into RT-QUIC (Fig 3, asterisk). This combination of dilutions is used for sPMCA/RT-QuIC throughout the remainder of this publication. Negative controls run with each dilution remained below the threshold and were combined for ease of graphics (Fig 3).

### Prion detection in brain tissue harvested from CWD+ CBP6 mouse bioassay

The use of conventional and amplification assays has contributed much to our current understanding of amyloid disorders, including prions. Yet, a limitation of these assays is their inability to directly measure infectivity. Bioassay remains the gold standard to assess infectivity. To determine the role *in vitro* analysis may have in providing insight to prion infectivity we compared results from five of these methods. To this end, brain tissue harvested from a dilutional mouse bioassay of CBP6 were assessed by conventional (IHC, WB) and amplification (sPMCA, RT-QuIC and sPMCA/RT-QuIC) assays. Insufficient quantities of mouse brain tissue negated inclusion of these samples for BioRad ELISA analysis.

#### Survival and detection of PrP^Sc^ by immunohistochemistry or western blot

**Fig 4:**
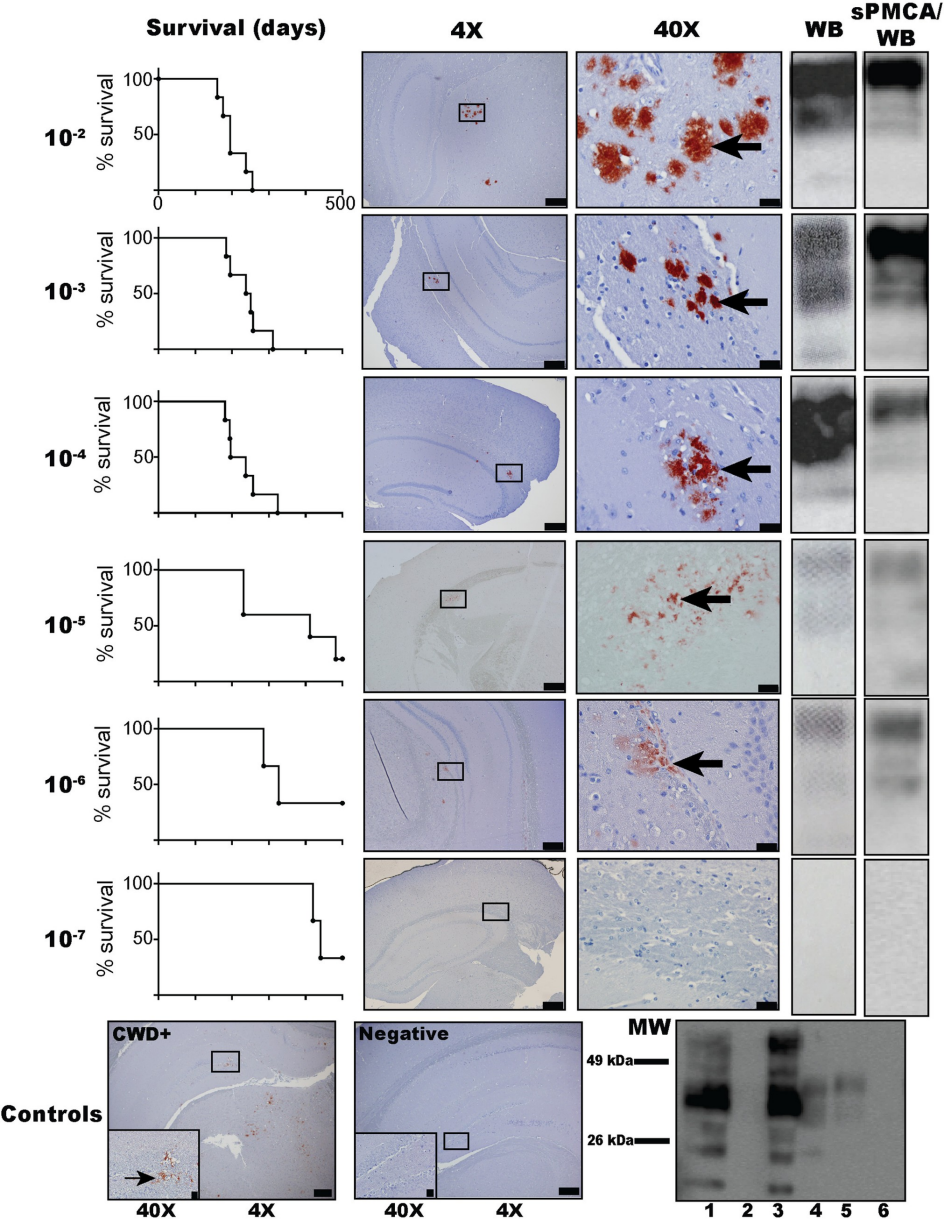
Dilutional mouse bioassay of CWD+ cervid brain into Tg(CerPrP) 5037 mice: survival curves, IHC, WB, and sPMCA/WB prion detection. PrP^Sc^ is shown in the brains of mice inoculated with dilutions 10^−2^–10^−6^ of CBP6 (300 μg-30 ng) using IHC (red deposits indicated with arrows), WB, and sPMCA/WB (5^th^ round product) (S1–S3 Figs). PrP^Sc^ was not detected in mice inoculated with the 10^−7^ CBP6 dilution (3 ng) nor in n = 12 negative control mice inoculated with naïve cervid brain. Bottom panels (left to right) show IHC PrP^Sc^ deposition in the positive control mouse brain (IC-inoculated 30 μl 10% CWD+ cervid brain homogenate) and no deposition in the negative control mouse (IC-inoculated 30 μl 10% naïve cervid brain homogenate). Control western blot (bottom right) shows complete PK digestion (50 μg/ml) of PrP^c^ in the unamplified (lane 2; without PK in lane 1) and amplified (lane 6) negative control mouse (IC-inoculated 30 μl 10% naïve cervid brain homogenate). PrP^Sc^ is revealed after PK digestion (50 μg/ml) in unamplified and sPMCA amplified CWD+ mouse brain (IC-inoculated with 30 μl 10% CWD+ cervid brain homogenate) (lanes 4 and 5; unamplified sample without PK in lane 3). IHC image objectives are 4X (scale bar = 200 μm) and 40X (scale bar = 20 μm).

A dilutional series mouse bioassay was conducted using CBP6 [36]. Six mice were intracranially-inoculated for each dilution (10^−2^–10^−7^). Two mice (one each from the 10^−5^ and 10^−6^ cohorts) were removed from the study due to death from causes other than prion infection. We found that all mice (11/11; 5-6/cohort) that were inoculated with 10^−2^ or 10^−3^ CWD+ cervid brain CBP6 succumbed to disease after an average of 203 (range 160 to 255) or 239 (184 to 257) days post inoculation (DPI), respectively (Table 2). PrP^Sc^ deposition was confirmed in all eleven mice by WB and IHC (Fig 4, S1 and S2 Figs). Five of six (5/6) mice inoculated with 10^−4^ CWD+ cervid brain CBP6 (average clinical disease 232 DPI, range 181 to 257), three of five (3/5) mice inoculated with 10^−5^ (average clinical disease 332 DPI, range 231 to 502) and 2/5 mice inoculated with 10^−6^ (average clinical disease 426 DPI, range 286 to 502) CWD+ cervid brain CBP6 showed signs consistent with prion disease and demonstrated PrP^Sc^ deposition by IHC and WB (Fig 4, Table 2, S1 and S2 Figs). Neither WB nor IHC identified PrP^Sc^ deposition in brain tissue of the six mice (0/6) inoculated with 10^−7^ CWD+ cervid brain CBP6 (502 DPI), nor mice (0/12) receiving naïve deer brain inoculum (502 DPI) (Fig 4, Table 2, S1 and S2 Figs).

**Table 2:** Prion detection in tissue harvested from a mouse dilutional bioassay of CWD+ cervid brain.

		Mouse cohort (average dpi to terminal disease ± SD) n+/total n						
Tissue tested	Assay	10^−2^ CWD (203±36)	10^−3^ CWD (239±46)	10^−4^ CWD (232±54)	10^−5^ CWD (372±133)	10^−6^ CWD (423±108)	10^−7^ CWD (>450)	Neg (NA)
**Brain**	**WB**	**6/6**	**6/6**	**5/6**	**2/5**	**2/5**	**0/6**	**0/12**
	**IHC**	**6/6**	**6/6**	**5/6**	**3/5**	**2/5**	**0/6**	**0/12**
	**sPMCA/** **WB**	**5/5**	**6/6**	**5/6**	**2/5**	**2/5**	**0/6**	**0/12**
	**RT-QuIC**	**5/5**	**6/6**	**5/6**	**3/5**	**2/5**	**0/6**	**0/12**
	**sPMCA/** **RT-QuIC**	**5/5**	**6/6**	**6/6**	**3/5**	**2/5**	**0/6**	**0/12**
**Spleen**	**RT-QuIC**	**5/5**	**6/6**	**5/6**	**3/5**	**2/5**	**0/6**	**0/12**

Comparison of *in vitro* conventional (WB, IHC) and amplification (RT-QuIC, sPMCA/WB and sPMCA/RT-QuIC) assays. Prions were detected in the brains of mice inoculated with CWD+ cervid brain (CBP6) dilutions 10^−2^–10^−6^ (300 μg-30 ng) by all assays. sPMCA/RT-QuIC detected prions in brain tissue of one additional mouse in the 10^−4^ cohort (3 μg seed material). In the 10^−5^ cohort, IHC, RT-QuIC, sPMCA/RT-QuIC and RT-QuIC of spleen tissue revealed one additional prion+ mouse than WB and sPMCA/WB. Prions were not detected in mice inoculated with the 10^−7^ CBP6 dilution (3 ng) nor in n = 12 negative control mice inoculated with naïve cervid brain homogenate.

#### Detection of amyloid seeding activity by RT-QuIC, sPMCA/WB, sPMCA/RT-QuIC

**Fig 5:**
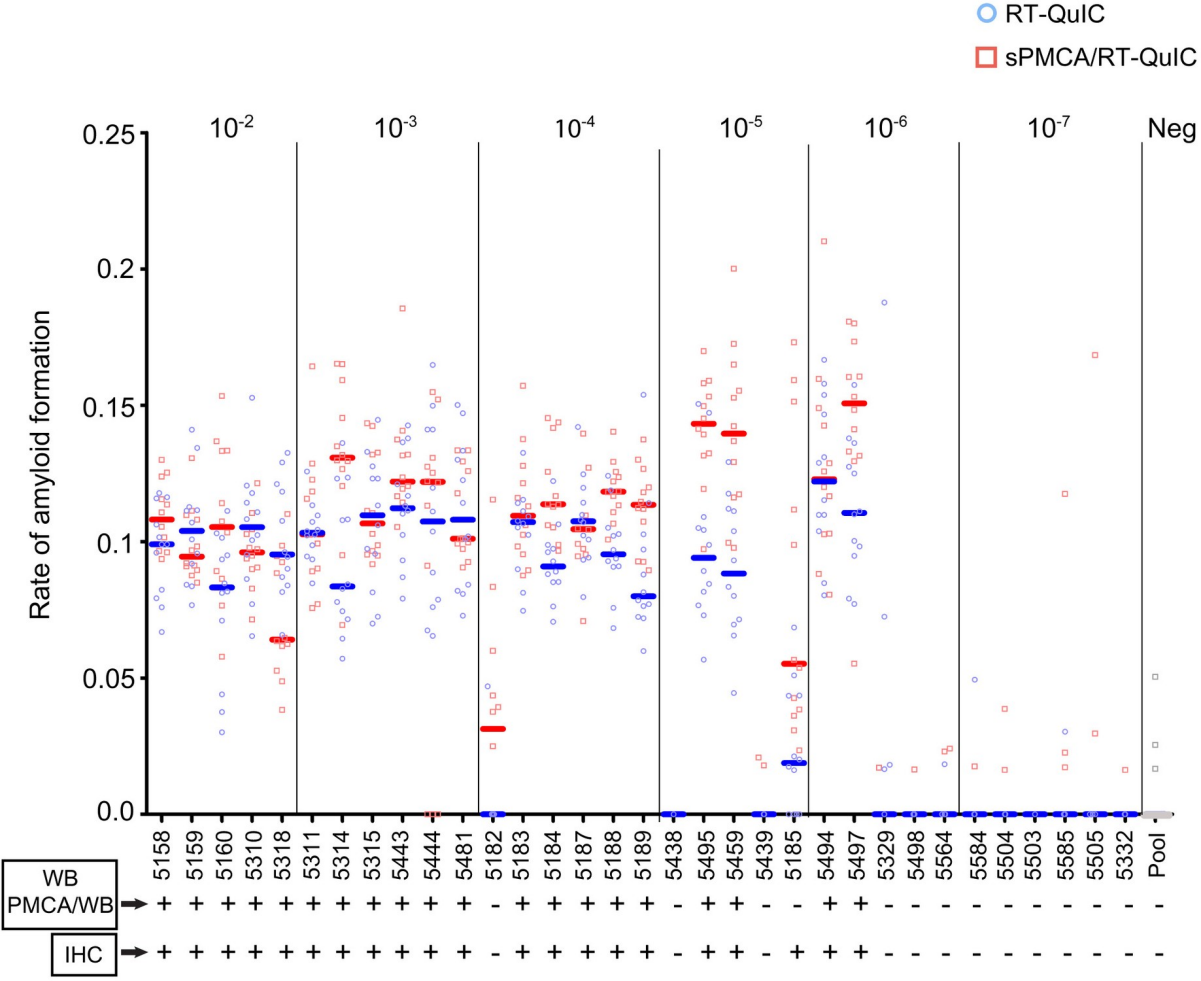
Prion detection in brain tissue harvested from a mouse dilutional bioassay of CWD+ cervid brain pool. Comparison of *in vitro* conventional (WB, IHC) and amplification (RT-QuIC, sPMCA/WB and sPMCA/RT-QuIC) assays. Readout for WB and PMCA/WB were the same for all mice (S2 and S3 Figs). IHC (S1 Fig), RT-QuIC and sPMCA/RT-QuIC detected prions in one additional mouse at 10^−5^ dilution of CBP6 (300ng seed material) than WB or PMCA/WB. RT-QuIC and sPMCA/RT-QuIC similarly detected prions in mice inoculated with 10^−2^, 10^−3^, 10^−5^ and 10^−6^ dilutions of CBP6 (300 μg, 30 μg, 300ng and 30 ng seed material; blue circles and median lines and red squares and median lines respectively). sPMCA/RT-QuIC detected prions in one additional mouse at 10^−4^ dilution of CBP6 (3 μg seed material). No assay detected prions in mice inoculated with the 10^−7^ dilution of CBP6 (3 ng seed material) or in negative control mice inoculated with naïve cervid brain (n = 12). Amplification assays represent 12 replicates/mouse/assay.

To further this cross-platform analysis brain tissue harvested from mice in the above dilutional bioassay were assessed by RT-QuIC and sPMCA. RT-QuIC, sPMCA/WB and sPMCA/RT-QuIC equally demonstrate amyloid seeding activity in the brain tissue of all eleven (11/11) mice receiving the highest concentration of CWD+ cervid brain (5-6/cohort; 10^−2^ and 10^−3^) (Fig 5, Table 2, S3 Fig). Insufficient tissue from one mouse in the 10^−2^ cohort precluded analysis for seeding activity by sPMCA and RT-QuIC. Upon analysis of brain tissue from mice receiving an intermediate dose of CWD+ cervid brain (10^−4^ cohort), both RT-QuIC and sPMCA/WB demonstrate amyloid seeding activity in 5/6 mice (Fig 5, Table 2, S3 Fig). Employing sPMCA/RT-QuIC we revealed amyloid seeding activity in all six (6/6) mice in the intermediate dose cohort (10^−4^) (Fig 5, Table 2). RT-QuIC analysis of brain tissue from bioassay mice receiving even lower doses of CWD+ cervid brain resulted in the detection of prion seeding activity in 3/5 mice (10^−5^ cohort), 2/5 mice (10^−6^ cohort) and 0/5 mice (10^−7^ cohort) (Fig 5, Table 2). sPMCA/RT-QuIC improved the amyloid conversion rates but did not increase the overall number of prion positive mice in cohorts 10^−5^–10^−7^ (Fig 5, Table 2). Negative assay controls and naïve mouse brain remained negative (Fig 5, Table 2).

#### Detection of additional prion-infected mice when assessed by RT-QuIC and sPMCA/RT-QuIC

Amyloid seeding activity was detected in the brain tissue of additional mice (5182, 5185) when assessed by RT-QuIC and sPMCA/RT-QuIC, but not by sPMCA/WB. Both of these mice had received a CWD+ cervid brain CBP6 dose in the LD_50_ range of the bioassay (10^−5.5^) (Fig 5, Table 2). To determine if the 11 CWD-inoculated yet brain negative mice, 5/11 in cohorts 10^−5^ and 10^−6^, the LD_50_ range, (5329, 5438, 5439, 5498 and 5564) and 6/11 in cohort 10^−7^ dilution cohort (5584, 5504, 5503, 5585, 5505, 5332) were not subclinical carriers we assessed spleen tissue by RT-QuIC (Fig 5, Table 2). Amyloid seeding activity was not detected in the spleen of any (0/11) of these mice (Table 2).

## Discussion

### RT-QuIC and sPMCA/QuIC amplification assays detect amyloid conversion beyond mouse bioassay LD_50_

The incorporation of amplification assays, sPMCA and RT-QuIC, enhanced the detection of prions in a CWD+ cervid brain pool CBP6 to levels beyond the mouse bioassay LD_50_ (Fig 4, Table 1). This may be due to a threshold requirement (minimum infectious dose: MID) to initiate infectivity *in vivo*. The existence of a MID threshold for viral and bacterial infectious agents has been observed [55]. While a small dose of viral or bacterial antigen may initiate a vaccinating immune response (live attenuated vaccine strategies), to initiate infection and disease progression a larger dose of pathogen is required. For prions this may be reflected by the initial success within the endoplasmic reticulum (ER) to activate the unfolded protein response (UPR) in an attempt to restore normal protein folding and ER function [56, 57], resulting in prolonged development of sufficient accumulation of amyloid required to initiate prion disease. Alternatively, these findings may suggest that a fraction of conversion competent amyloid, as detected by *in vitro* amplification assays, is noninfectious. Previous studies have demonstrated that both infectious and noninfectious amyloid particles exist in prion inoculums [58]. It is also possible that this attests to the *in vitro* amplification process being more efficient at initiating amyloid conversion events than its *in vivo* counterpart.

### sPMCA and RT-QuIC amyloid seeding activity is inhibited at high seed concentrations and rescued by dilution

We confirm earlier reports [36, 54, 59] that sPMCA and RT-QuIC amyloid seeding activity remains undetectable in tissue and biological fluid samples when spiked into either assay at high concentrations (10^−1^–10^−2^ dilution) (Figs 2 and 3). Here, employing the CWD+ cervid brain pool CBP6, we demonstrate that amyloid detection in sPMCA product by RT-QuIC is rescued and provides peak detection when dilutions for both sPMCA and RT-QuIC are optimized (Fig 2). Observed inhibition may be related to components or matrix effects that occur in less dilute samples. Optimization may be required when assessing seeding activity by single or combined use of amplification assays to account for variation in amyloid conversion rates dependent upon tissue/fluid type, host species, prion strain or PRNP genotype.

### Amplification assays detect prions in terminal brain tissue with similar efficacy as conventional IHC

We found that conventional IHC detected prions (PrP^Sc^ deposition) in mouse bioassay terminal brain tissue with similar efficacy as the new generation of amplification assays (prion seeding activity) (Figs 4 and 5, S1–S3 Figs, and Table 2). These findings affirm the robust nature of conventional test assays and supports their continued use in the application of testing tissues harvested from terminal, end stage disease cases, when PrP^Sc^ levels are high. The detection of high concentrations of prions by both conventional and amplification assays has been previously substantiated in tissues harvested during the late and terminal phases of prion disease [60–64]. Of increasing interest is the ability to detect very low concentrations of prions associated with early infection and silent carriers of disease. Conventional assays have been used to detect prions in lymphoid biopsies harvested from hosts lacking signs/symptoms of disease [65–67]. Yet, these methods present concerns regarding diagnostic accuracy due to limitations associated with the amount of tissue that can be analyzed, the low detection capacity inherent to these methods, and by the presence of protease sensitive prion isoforms that may be abolished by PK digestion [68].

### New generation in vitro amplification assays provide improved detection rigor

We demonstrate that amplification assays can improve detection rigor as prion burden decreases (Figs 3 and 5). The ability to assess 8–16 replicates from a single sample improves readout reliability, and thus provides a screening mechanism for the detection of very low concentrations of prions present shortly after prion exposure, or for the assessment of nonclassical and asymptomatic disease states. These findings are supported by previous studies that have employed these techniques to demonstrate prions in samples harvested at very early time points post experimental infection or during the asymptomatic phase of disease [6, 18, 38, 40, 43, 44, 50, 69–72].

### In vitro amplification assays provide enhanced detection of prion infection at the mouse bioassay LD_50_

As mentioned above, our study finds that *in vitro* amplification by RT-QuIC and sPMCA/RT-QuIC demonstrate enhanced detection of seeding activity in a CWD+ cervid brain pool homogenate than that reached by conventional test results from mouse bioassay of the same homogenate. Further analysis of terminal mouse brain tissue from the dilutional bioassay by RT-QuIC and sPMCA revealed 2 additional prion-infected mice at the mouse bioassay LD_50_ (Fig 5). These findings further corroborate the published LD_50_ established for this CWD+ brain pool [36]. Interestingly, use of sPMCA/RT-QuIC revealed an increase in amyloid seeding rates for most of the bioassay mice (Fig 5). Both findings may have impact upon earlier detection and diagnosis of disease status, and prion detection in samples containing minute quantities of amyloid seeding activity. Our findings are isolated to the detection of prions in brain tissues and a single CWD+ brain pool, but are supported by a recent report [52] where the use of sPMCA/RT-QuIC demonstrate enhanced detection of seeding activity in biological fluids containing very low prion burdens. These findings may be especially important for antemortem prion diagnosis. Bioassay remains the gold standard and definitive readout for the detection of prion infectivity. Its use has provided tremendous insight to our understanding of prion pathogenesis and transmission dynamics [3, 71, 73–78]. Yet, it is costly in money, time and animal use. Bioassay of prion concentrations near the LD_50_ requires the use of 9–12 animals/cohort and between 400–500 days to reach a study endpoint. Conservatively, adding in animal costs, per diem and assessment of tissue, this equates to thousands of dollars and 1–2 years.

### Future use for new generation amplification assays

This new generation of *in vitro* amplification assays have been reported to obtain sensitivity levels rivaling that of bioassay [27, 79]. Our results confirm that sPMCA and RT-QuIC detect prions to bioassay endpoint (Fig 5, Table 2). We also show that RT-QuIC can detect additional seeding activity not demonstrated when sPMCA is analyzed by WB. Prion infectivity is known to consist of variably protease sensitive/resistant isoforms [80]. RT-QuIC avoids the use of proteinase K, and thus, may enhance detection of protease sensitive isoforms. While more testing is required, we propose that RT-QuIC and sPMCA/RT-QuIC may be useful to assess prion infectivity instead of bioassay.

Recent evidence suggests that other neurodegenerative diseases such as Parkinson’s Disease (PD), Alzheimer’s Disease (AD), and Huntington’s disease produce prion-like aggregates [81]. Studies have utilized sPMCA [82–84] and RT-QuIC [30, 84] to detect α-synuclein proteins and β-amyloid oligomers in cerebral spinal fluid of AD and PD patients, respectively. Modified PMCA is able to detect polymers with high sensitivity and specificity, and has the capacity to distinguish these neurodegenerative diseases from others [82]. Similar to prion diseases, PD, AD and other neurodegenerative diseases have a long latency period during which time detection is limited. Current diagnostic methods for these human protein misfolding disorders rely upon imaging techniques, clinical examination and screening for potential biomarkers, however, a definitive diagnosis can only be made from post-mortem histology [85]. Earlier diagnosis will be key to continued development of therapeutics for all neurodegenerative diseases.

In summary, we provide evidence that the use of ‘new generation’ amplification assays enhance prion detection sensitivity and improve test validity and rigor for the analysis of biological samples containing very low concentrations of prions. Methods employing *in vitro* detection will never replace bioassay, but they can augment findings and become tools for the early diagnosis of human and animal protein misfolding disorders.

## Materials and methods

### Ethics statement

All animals were handled in strict accordance with guidelines for animal care and use provided by the United States Department of Agriculture (USDA), National Institutes of Health (NIH) and the Association for Assessment and Accreditation of Laboratory Animal Care International (AAALAC), and all animal work was approved by Colorado State University Institutional Animal Care and Use Committee (IACUC protocol numbers 10-2189A, 12-3773A, and 13-4482A).

#### Cervids handling

Naïve deer brain homogenate: Naïve brain homogenate derived from pooling the brain tissue harvested from two naïve and IHC confirmed CWD-free white-tailed deer served as a negative control. Hereafter referred to as naïve cervid brain.

CWD-infected deer brain homogenates (CBP6): CWD positive inoculum was derived from pooling the brain tissue harvested from six terminal CWD-infected and IHC-confirmed positive white-tailed deer (CBP6) generated from previous experimental studies conducted at CSU [86, 87]. The inoculum is hereafter referred to as CWD+ cervid brain CBP6. The deer were acquired from Warnell School of Forestry, University of Georgia-Athens. They were housed socially in 6×12 meter suites with sand and epoxy mixture sealed floors to afford normal wear on hooves. The sealed floors were covered in a layer of aspen chip bedding. A natural light equivalent was mimicked, humidity was maintained at 25–40%, and temperature ranged from 16–26 degrees Celsius. Enrichment consisted of cardboard boxes, ropes and plastic toys, and daily interaction with caretaker personnel. Deer received 50 g complete pelleted feed per kg/day along with hay forage and water ad libitum. For sample collection (including prior to euthanasia), deer were anesthetized with IM injections of Ketamine (2–8 mg/kg) and Medetomidine (0.1–0.2 mg/kg). Euthanasia was performed by IV injection of pentobarbital sodium with phenytoin (1 ml per 4.5 kg).

#### Mice handling

Naïve mouse brain homogenate: Brain homogenate from transgenic mice overexpressing the elk prion protein, Tg(CerPrP) 5037, [88] inoculated with naïve cervid brain served as a negative control for all assays.

Bioassay mouse brain tissues: Tg(CerPrP) 5037 mice were used to establish an LD_50_ of CBP6 CWD+ cervid brain [36]. Mice were housed socially in a commercial caging system in cages filled with aspen chips, provided nesting materials, checked daily, and provided commercial irradiated rodent chow and water ad libitum. Prior to inoculation, mice are given a 2 mg/kg dose of meloxicam analgesic. Briefly, mouse cohorts (n = 6/cohort) were inoculated intracranially in the right parietal lobe of the cerebral cortex with 30 μl of 10^−1^–10^−7^ dilutions of CBP6 and were observed for signs of prion disease. Mice were terminated by inhalation of CO_2_ upon development of prion disease or 500 days post infection (dpi). Brain tissue harvested from each inoculated mouse was divided in half with one half frozen at -80°C and one half fixed in periodate-lysine-paraformaldehyde (PLP) fixative for a minimum of 48 h. After a minimum of 48 h fixed tissues were transferred to 1xPBS at room temperature until further analysis.

### Western blot (WB)

Brain tissue from Tg(CerPrP) 5037-inoculated mice was prepared as a 10% (w/v) homogenate in 1xPBS and stored at 4°C until further analysis. Homogenates were mixed with proteinase K (PK; Invitrogen) at 50 μg/mL, incubated at 37°C for 30 min followed by 45°C for 10 min. Samples were mixed with reducing agent (10x)—lithium dodecyl sulfate (LDS) sample buffer (4x) (Invitrogen) at a concentration of 1x, heated at 95°C for 5 min and separated on NuPAGE 12% Bis-Tris gel at 125V for 1.5 h. Protein was transferred to a polyvinylidene fluoride (PVDF) membrane at 80 V for 1 h in transfer buffer (0.025 M Trizma base, 0.2 M glycine, 20% methanol, pH 8.3). The membrane was then incubated with 5% nonfat milk in 1xTris-buffered saline (TBS) with 0.1% Tween 20 (TBST) for 3 min and then for 12 min with BAR224-HRP (0.2 μg/ml final concentration; Cayman Chemical) diluted in TBST, followed by a 30 min wash with TBST. The membrane was then developed with ECL Plus Western blotting detection reagents (Pierce) and viewed on a Luminescent Image Analyzer LAS-4000 (Fujifilm).

### BioRad enzyme-linked immunosorbant assay (ELISA)

Brain tissue dilutional series (10^−1^–10^−9^) of naïve and CWD+ cervid brain homogenate (CBP6) were performed at the Colorado State University Diagnostic Laboratory as per BioRad instructions. The read-out criteria used were Suspect (OD ≥ 0.116), Questionable (OD 0.094–0.115) or Not Detected (< 0.093).

### Immunohistochemistry (IHC)

PLP fixed mouse brains were paraffin-embedded and 5 μm tissue sections were cut and mounted on glass positive charge slides. Tissues were deparaffinized in a 65°C oven followed by successive xylene immersions (100%), rehydrated through graded ethanol washes (100% x 2, 95% x 2 and 70% at 5 min per wash), and 88% formic acid treated for 60 min. Tissues were then subjected to hydrated autoclaving using an automated antigen-retrieval system 2100-Retriever (Prestige Medical) and a citrate buffer (0.01M sodium citrate, 0.05% tween 20, pH 6) for 30 min. Samples were then blocked with 3% H_2_O_2_ (30 min) followed by a proprietary protein block (TNB, PerkinElmer Life and Analytical Sciences) (30 min) and stained with unconjugated BAR-224 at 2 μg/ml (Cayman Chemical) (overnight at 4°C). Detection was completed using HRP-conjugated anti-mouse secondary antibody (Envision+, Dako) (30 min) and AEC substrate chromogen (Dako) (1 min). Tissues were then counterstained with Meyer’s hematoxylin (Dako) (2 min), followed by 0.1% calcium bicarbonate bluing reagent (5 min) and coverslipped with aqueous mounting media (Dako).

### Serial protein misfolding cyclic amplification (sPMCA)

Tg(CerPrP) 5037normal brain homogenate (NBH): NBH, the PrP^C^ substrate used for sPMCA prion conversion, was prepared as follows: naïve Tg(CerPrP) 5037 mice <4 months of age were euthanized by CO_2_ inhalation and perfused with 35 mL of 5 mM ethylenediaminetetraacetic acid tetrasodium salt (EDTA) in PBS via intracardiac catheterization. The brain was removed and flash frozen using liquid nitrogen. Brain homogenate was then prepared at a 10% (w/v) solution in sPMCA buffer (1% Triton-X 100 [v/v], 5 mM EDTA, and 150 mM NaCl) with the addition of Complete Protease Inhibitors (Roche Pharmaceuticals, Indianapolis, IN) in a homogenizer (Omni Bead Ruptor). Homogenates were then centrifuged for 1 min at 3000 rpm to remove bulk brain material, and the supernatant frozen in single-experiment aliquots at -80°C in a prion-free room until use in sPMCA.

sPMCA: Thirty microliters (30 μl) of each sample (3 replicates for each sample, 2 investigators/sample; investigators were blinded to sample identity) was spiked into 50 μl of NBH and sonicated for 10 sec every 5 min for each 24 h round. After each 24 h round 30 μl of sonicated material was transferred into 50 μl of fresh NBH and subjected to another 24 h round for a total of 5 rounds. After 5 rounds material was analyzed by WB as described previously [71]. Naïve and CWD+ cervid brain inoculum (CBP6) served as positive and negative sPMCA controls.

### Real-time quaking induced conversion assay (RT-QuIC)

Substrate for seeded RT-QuIC reactions was prepared by adding truncated Syrian Hamster recombinant protein encoding residues 90–231 (SHrPrP) prepared as previously reported [38] to RT-QuIC buffer (320 mM NaCl, 1.0 mM EDTA, 10 μM Thioflavin T [ThT, Sigma]) at a final concentration of 0.1 mg/ml. Ninety-eight microliters (98 μl) RT-QuIC substrate was added to optical bottom black 96-well plates (Nunc). Each mouse brain homogenate from the mouse CBP6 bioassay was diluted 10^−2^ to 10^−7^, and 2 μl of each was run in quadruplicate on 2–3 plates by 2–3 investigators for a total of 7–12 replicates/sample. Positive and negative controls were included on all plates. Prepared plates were placed in a BMG Labtech Polarstar fluorometer and subjected to 700 rpm double-orbital shaking for 1 min ever other min for 15 min for 250 cycles. After each cycle ThT fluorescence was read at an excitation of 450 nm and emission of 480 nm. Gain was set at 1700 and read using orbital averaging with 20 flashes per well with the 4 mm setting. Fluorescent readings were recorded for all sample reactions for a total time of 62 h at a temperature of 42°C. Samples were considered positive if they crossed a threshold (5 SD above the mean of the initial 5 readings). The inverse of the time when the reaction reached the threshold (1/time to threshold) was then used to determine the amyloid formation rate. Statistical analyses were run in Prism v6, GraphPad Software, La Jolla, CA. A Mann-Whitney test was used to generate (p-values less than 0.05 were considered significant) by comparing the sample rates to the rates of known negative control tissues. A Reed-Muench calculation was used to calculate the LD_50_ for the titrated mouse bioassay as previously described [36].

### RT-QuIC readout of sPMCA material (sPMCA/RT-QuIC)

sPMCA 5^th^ round product was diluted 1:100 or 1:1000 in 0.1% SDS/PBS (see figure legends for sample dilution). Two microliters (2 μl) of each sample was analyzed by RT-QuIC as described above. The rates were calculated and graphed as previously described [52].

## Supporting information

S1 Dataset Rates of amyloid formation (1/time to threshold) used to create datapoints presented in Fig 2. (XLSX)

S2 Dataset Rates of amyloid formation (1/time to threshold) used to create datapoints presented in Fig 3. (XLSX)

S3 Dataset Rates of amyloid formation (1/time to threshold) used to create datapoints presented in Fig 5. (XLSX)

S1 Fig Immunohistochemistry (IHC) of brain tissue harvested from mouse dilutional bioassay of CWD+ cervid brain pool represented in Fig 5. (TIF)

S2 Fig Western blot (WB) analysis of brain tissue harvested from mouse dilutional bioassay of CWD+ cervid brain pool represented in Fig 5. (TIF)

S3 Fig Western blot (WB) analysis of product from 5^th^ round serial protein misfolding cyclic amplification (PMCA/WB) of brain tissue harvested from mouse dilutional bioassay of CWD+ cervid brain pool represented in Fig 5. (TIF)
