# Supplementary figures and images for: Comparison of conventional, amplification and bio-assay detection methods for a chronic wasting disease inoculum pool
Source: PLoS One. 2019 May 9;14(5):e0216621. doi: 10.1371/journal.pone.0216621 (PMC6508678; doi:10.1371/journal.pone.0216621)

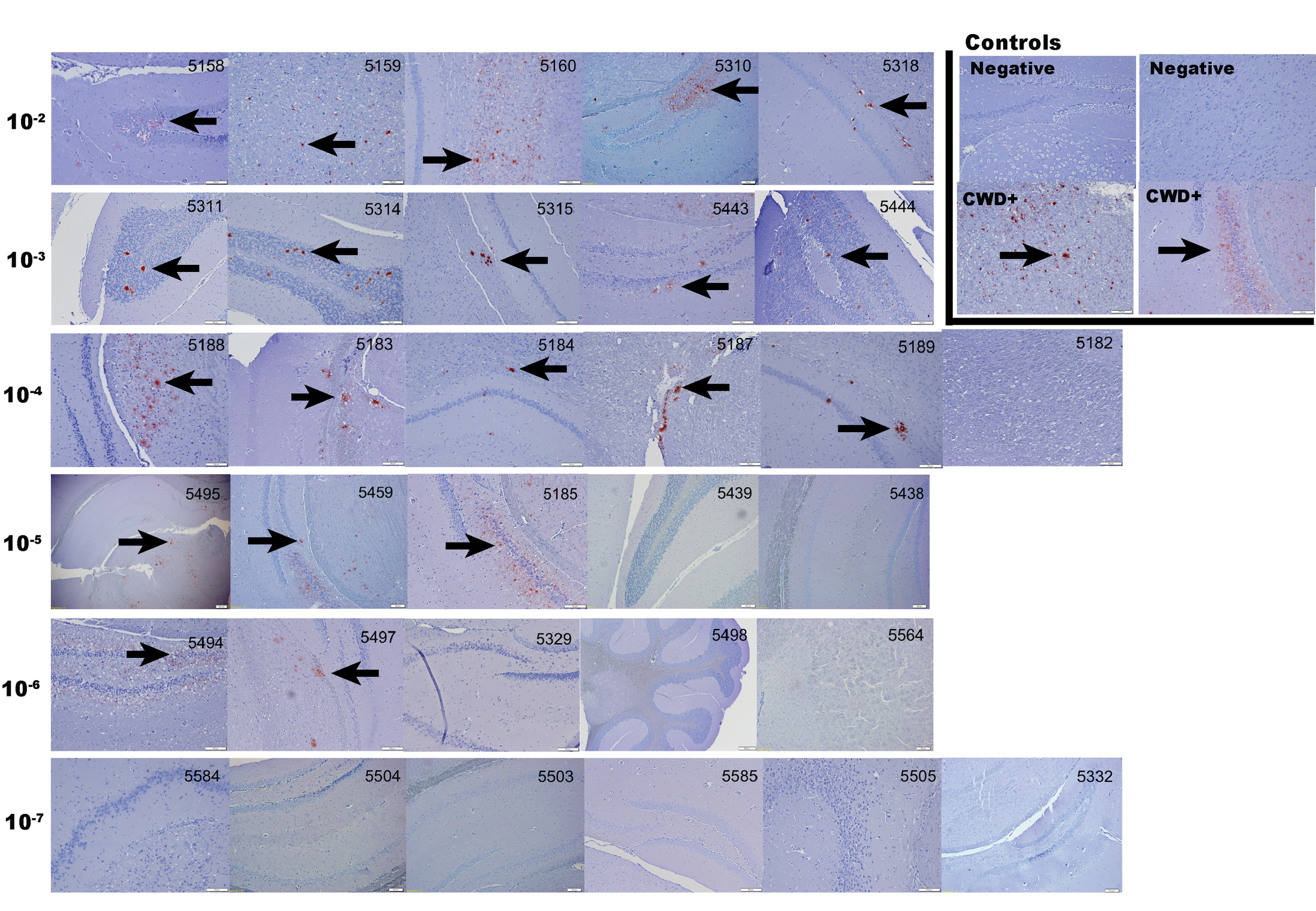

Supplement: S1 Fig — (TIF) [file pone.0216621.s004.tif]

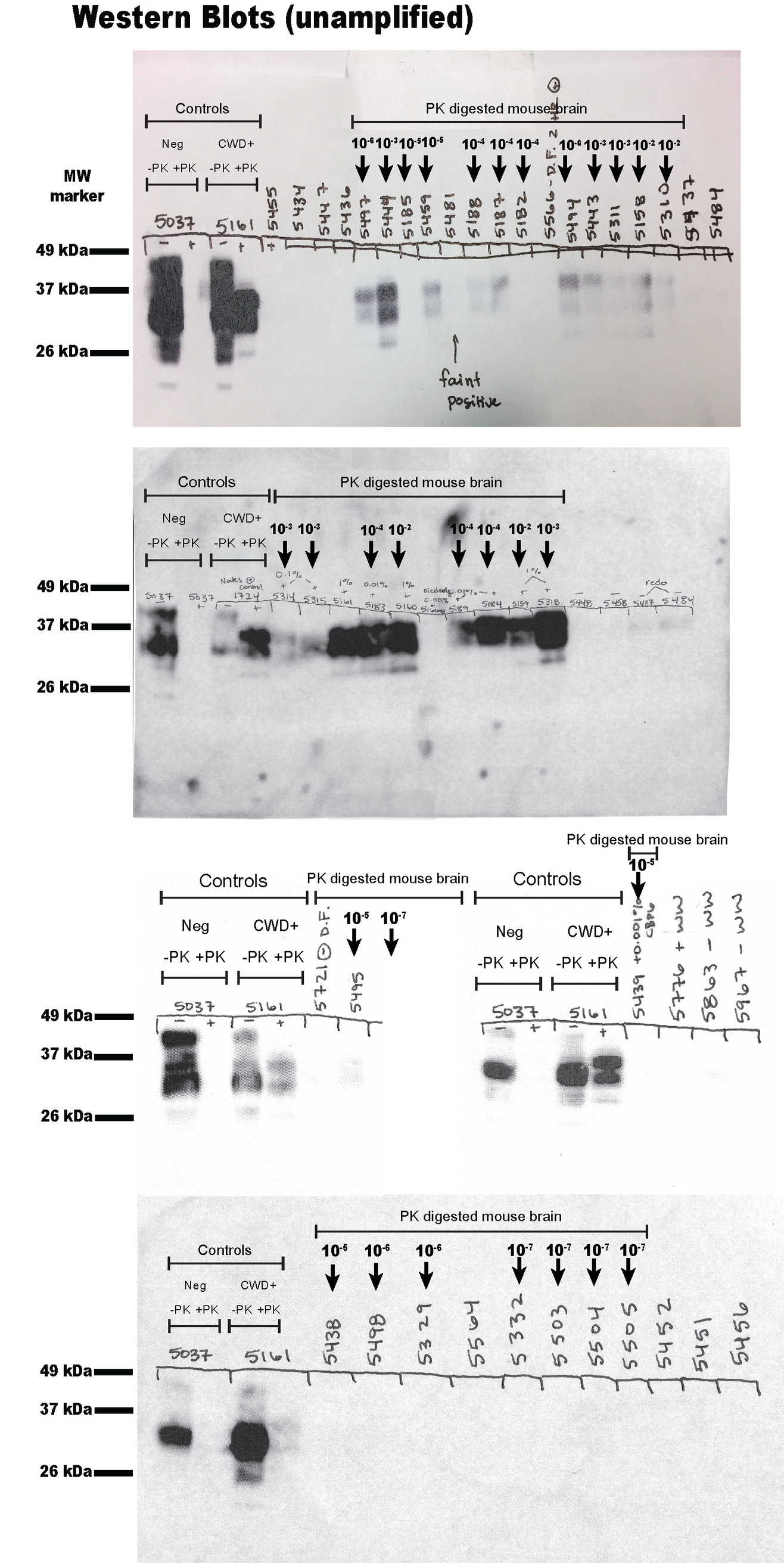

Supplement: S2 Fig — (TIF) [file pone.0216621.s005.tif]

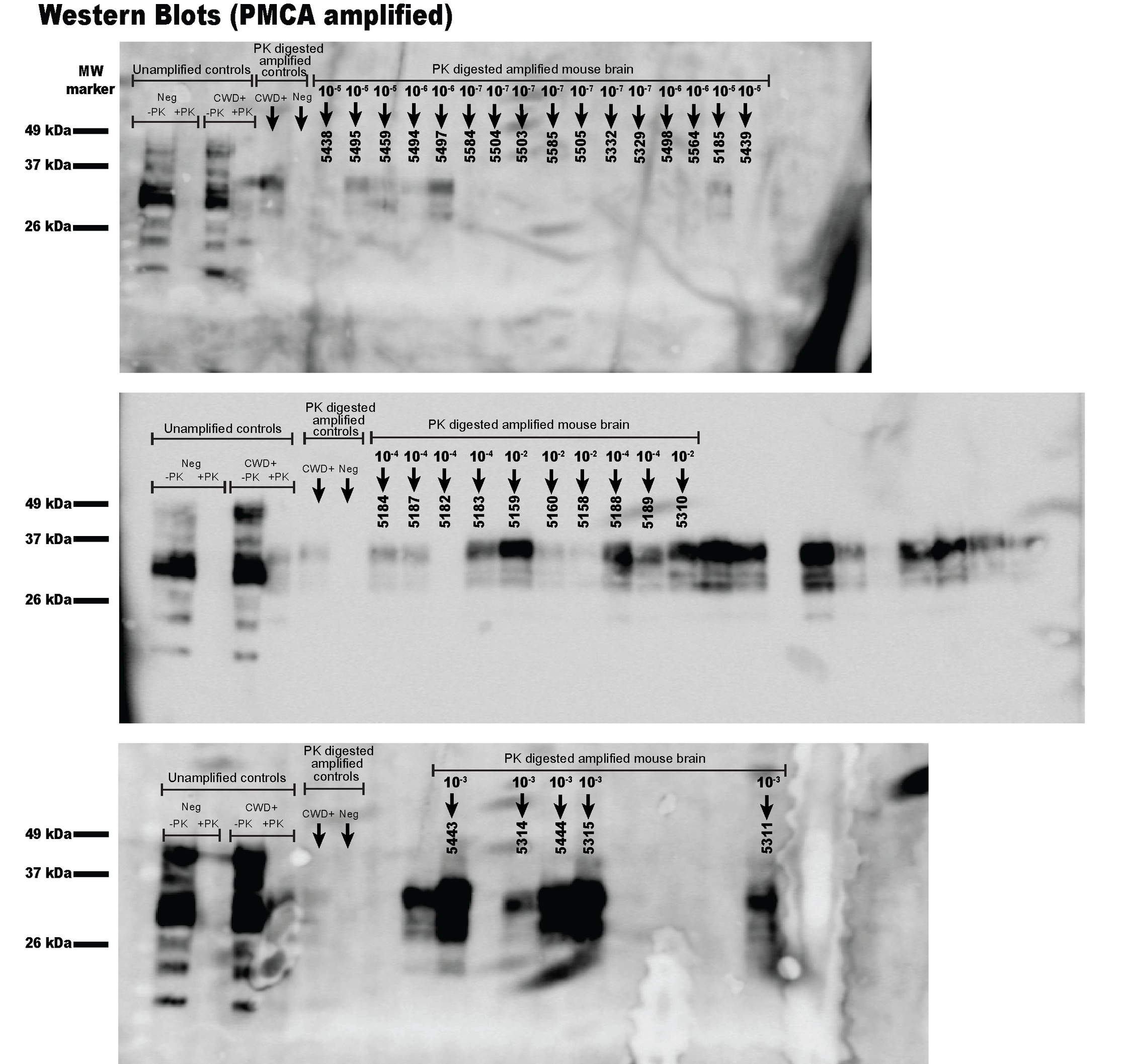

Supplement: S3 Fig — (TIF) [file pone.0216621.s006.tif]
